# Follow-up of intramyocardial bone marrow mononuclear cell transplantation beyond 10 years

**DOI:** 10.1038/s41598-024-53776-9

**Published:** 2024-02-14

**Authors:** Severi Mulari, Risto Kesävuori, Juhani A. Stewart, Pasi Karjalainen, Miia Holmström, Miia Lehtinen, Juha Peltonen, Mika Laine, Juha Sinisalo, Tatu Juvonen, Markku Kupari, Ari Harjula, Tommi Pätilä, Sari Kivistö, Esko Kankuri, Antti Vento, the Helsinki BMMC Collaboration, Pekka Hämmäinen, Jukka Schildt, Aapo Ahonen, Päivi Nikkinen, Anne Nihtinen, Riitta Alitalo, Reino Pöyhiä

**Affiliations:** 1 Heart and Lung Center, Helsinki University Hospital and University of Helsinki, Helsinki, Finland; 2 Faculty of Medicine, Department of Pharmacology, University of Helsinki, Haartmaninkatu 8, PO Box 63, 00014 Helsinki, Finland; 3 Department of Radiology, HUS Medical Imaging Center and Helsinki University Hospital and University of Helsinki, Helsinki, Finland; 4 Pediatric Cardiac Surgery, Children’s Hospital, Helsinki University Hospital and University of Helsinki, Helsinki, Finland; 5 Department of Cardiothoracic Surgery, Heart and Lung Center, Helsinki University Central Hospital, Helsinki, Finland; 6 Division of Nuclear Medicine, HUS Medical Imaging Center, Helsinki University Central Hospital, Helsinki, Finland; 7 Department of Hematology, Helsinki University Central Hospital, Helsinki, Finland; 8 Stem Cell Laboratory, Department of Clinical Chemistry and Hematology, HUSLAB, Helsinki University Central Hospital, Helsinki, Finland; 9 Department of Anesthesiology and Intensive Care, Helsinki University Central Hospital, Helsinki, Finland

**Keywords:** Cardiology, Medical research

## Abstract

Bone marrow mononuclear cells (BMMCs) have been evaluated for their ability to improve cardiac repair and benefit patients with severe ischemic heart disease and heart failure. In our single-center trial in 2006–2011 we demonstrated the safety and efficacy of BMMCs injected intramyocardially in conjunction with coronary artery bypass surgery. The effect persisted in the follow-up study 5 years later. In this study, we investigated the efficacy of BMMC therapy beyond 10 years. A total of 18 patients (46%) died during over 10-years follow-up and 21 were contacted for participation. Late gadolinium enhancement cardiac magnetic resonance imaging (CMRI) and clinical evaluation were performed on 14 patients, seven from each group. CMRIs from the study baseline, 1-year and 5-years follow-ups were re-analyzed to enable comparison. The CMRI demonstrated a 2.1-fold larger reduction in the mass of late gadolinium enhancement values between the preoperative and the over 10-years follow-up, suggesting less scar or fibrosis after BMMC treatment (− 15.1%; 95% CI − 23 to − 6.7% vs. − 7.3%; 95% CI − 16 to 4.5%, *p* = 0.039), compared to placebo. No differences in mortality or morbidity were observed. Intramyocardially injected BMMCs may exert long-term benefits in patients with ischemic heart failure. This deserves further evaluation in patients who have received BMMCs in international clinical studies over two decades.

## Introduction

Myocardial transplantation of autologous bone marrow mononuclear cells (BMMCs) has been widely studied over two decades^1^. We^2,3^, and others^4^, have evaluated the effect of intramyocardial BMMC injections in ischemic heart failure (HF)^5^. The results demonstrate a significant reduction of myocardial scar volume. The effect is seen in segments receiving BMMCs, but not in segments receiving placebo injections. Follow-up studies have reached five to seven years and have reported promising results, but confirmation of the long-term persistence of treatment effects is lacking.

In their 2014 and 2016 Cochrane reviews, Fisher et al. concluded that bone marrow-derived cell treatments improved cardiac function up to five years of follow-up^6,7^. Moreover, they concluded that BMMC transplantation reduces mortality in HF patients^8^. This view was supported by Wang et al., who reported that stem cell transplantation in ischemic HF may contribute to the improvement of left ventricular ejection fraction (LVEF), as well as the reduction of the New York Heart Association (NYHA) functional HF classification, Canadian Cardiovascular Society Angina Score grade, and left ventricular end systolic volume (LVESV). According to their meta-analysis, stem cell transplantation did not affect mortality^9^. However, the meta-analysis by Lee et al. suggested that beneficial effects of cell therapy need to be evaluated by long-term follow-up outcomes^8–10^.

During 2006–2011 we conducted a randomized, double-blinded, and placebo-controlled clinical trial (ClinicalTrials.gov Identifier: NCT00418418) investigating the safety and efficacy of BMMC transplantation during coronary artery bypass graft (CABG) surgery. BMMC therapy or placebo were administered as 15–20 0.2-ml injections into predefined sites in the infarction border area during CABG surgery. Preoperative late gadolinium enhancement cardiac magnetic resonance imaging (CMRI) data were used for preoperative localization of the infarction area scar and its border areas. Both the BMMC and placebo injections were targeted based on that data to the ischemic scar border areas during CABG surgery. The initial follow-up was at 1 year. We showed that BMMC intramyocardial injections, but not placebo injections, significantly reduced myocardial scar size in the treated areas. The therapy was safe but had no effect on cardiac function at one year follow-up^2^. We also demonstrated the intracardiac injections to be safely administered during surgery without arrhythmic or other complications during the otherwise vulnerable postoperative stay at the intensive care unit^11^. Then, in 2013, we conducted a 5-years follow-up study of these patients. There was no major mortality in either the placebo or BMMC-treatment groups, but intriguingly the previously observed myocardial scar-reducing effect of BMMC therapy was sustained. The BMMC-treated myocardial segments showed a median change of − 17% (IQR, − 30 to − 6%) in scar size, whereas in the placebo-treated segments scar size increased by a median of 2% (IQR, − 7 to 19%)^3^.

As our BMMC study matured and the follow-up times for all patients reached 10 years or more, we designed a long-term follow-up study and invited all surviving study patients to a 10–14 years follow-up visit. As in the previous studies, left ventricle (LV) function and scar size were analyzed using late gadolinium enhancement CMRI. The main endpoints of follow-up were cardiac mortality and morbidity, as well as the myocardial changes in the BMMC- and placebo-treated segments as measured using CMRI. To ascertain the best possible comparability between the follow-up time points, we meticulously reanalyzed the previous CMRI images using the same current state-of-the-art methodology as used for the newly obtained sequences. In addition, we evaluated the patients’ six-minute walk test (6MWT) performance, measured the circulating pro-B-type amino-terminal natriuretic peptide (NT-ProBNP) concentrations, determined the NYHA functional classes, and used the Medical Outcomes Study Short-Form 36 (SF-36) Health Survey to assess the health-related quality of life (HRQoL).

## Methods

This study was approved by the Institutional Ethics Committee of the Hospital District of Helsinki and Uusimaa (Dnro HUS/3181/2019 and HUS 456/E6/05). All methods were performed in accordance with the relevant guidelines and regulations. All measurements and analyses were performed by researchers not involved in the previous studies (SM, RK, JAS, PK, and JP) and remaining blinded to the study groups.

### Patients

The original study protocol, one-year results and our previous follow-up study’s results have been published previously^2,3^. The original trial patients had to meet the following inclusion criteria: age between 18 and 75 years, provided informed consent, LVEF 15–45% and NYHA class II to IV HF symptoms. The exclusion criteria are presented in Supplementary Table 1.

**Figure 1:**
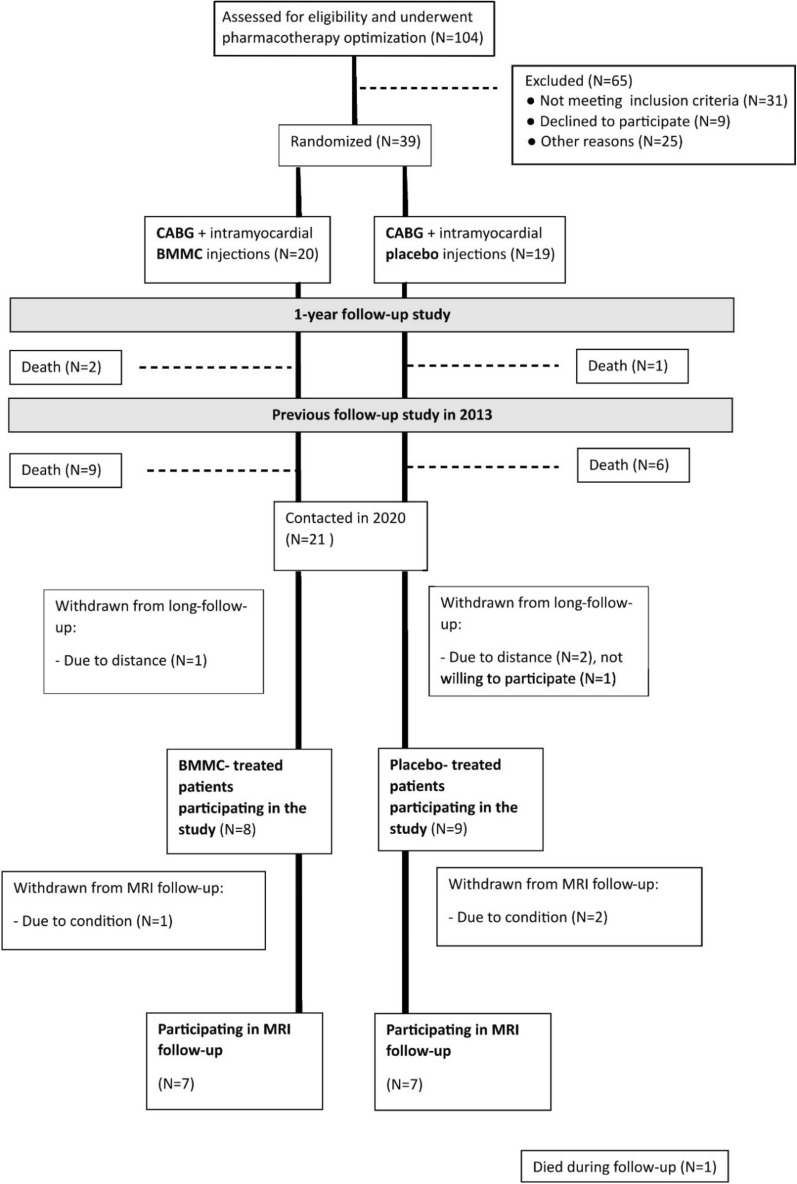
Flowchart presenting patients participating in over 10 years follow-up study.

A total of 39 patients were initially enrolled to the original study (Fig. 1). The enrolled patients’ HF medication was first optimized to meet contemporary best practice guidelines. After a 4-week period of drug optimization the screening echocardiogram was repeated. 39 patients (39/104, 37.5%) meeting the inclusion criteria after the drug optimization period were included into the study after informed consent, and CABG operation was scheduled. It is noteworthy that this type of pre-study optimization of HF medication is unique in its ability to select medical-treatment-refractory patients, receiving no additional benefit from optimization of HF medication strategies, into the study.

### BMMC or placebo injection

At the beginning of the surgery and after anesthesia induction, bone marrow aliquots (100 ml) were harvested from all patients from the posterior iliac crest. The standard methods of Helsinki University Meilahti Cell Processing Laboratory were used to obtain the mononuclear cell fraction^2^. The cell suspension was divided into six one-milliliter syringes for the treatment group; the placebo group received only vehicle medium by syringes. Patients were assigned randomly, and the syringe contents were masked to retain the double-blinded technique. Cell counts and flow cytometry of the bone marrow harvest were performed for the treatment group using standard methods from the clinical flow cytometry laboratory and FACSCalibur (Becton–Dickinson, San Jose, CA). The median count of BMMCs administered was 8.4 × 10^8^ (IQR, 5.2 × 10^8^ to 13.5 × 10^8^). Of these, 1.2% were CD34^+^, 0.9% CD34^+^CD133^+^ and 0.8% CD34^+^CD117^+^^2^.

A standard CABG operation was performed after the bone marrow aspiration. After the bypass anastomosis was done and the heart was still in asystole caused by cardioplegia, the BMMCs (15–20 0.2-ml injections) were injected into sites predefined for each individual patient separately, based on the preoperative CMRI data, in the infarction border area. Imaging data were used for preoperative localization of the infarct scar and its border areas. Both BMMC and placebo injections were guided by that data to target the injections to the infarct border area. The injection procedure was carefully photographed during the surgery, and the segments injected were specified in patient documents for later analysis.

### More than 10-year follow-up

After our previous follow-up study, patients were monitored in outpatient clinics in their home areas of the Helsinki Central Hospital District or in the regional health centers according to national clinical guidelines. In 2020, patients were invited for a more than 10-year follow-up visit into the Heart and Lung Center, Meilahti Hospital. For the end point measures in this long-term follow-up study we determined same measures as in our previous follow-up: (1) changes in LV measurements and changes in scar size, (2) concomitant changes in NYHA class and plasma NT-ProBNP concentration, (3) hospitalizations due to cardiac cause, (4) the patients’ current HRQoL by the SF-36 questionnaire. Moreover, in this long-term follow-up we also carried out evaluations of (5) mortality during the follow-up were obtained from the national population archive and from clinical databases, (6) patients’ clinical status done by cardiologist and 6MWT to screen physical condition, and (7) Major Adverse Cardiac and Cerebrovascular Events (MACCE) and Major Adverse Cardiac Events (MACE) were obtained between groups. MACCE was defined as all-cause mortality, stroke or transient ischemic attack, nonfatal myocardial infarction, acute coronary syndrome (including unstable angina), and hospitalization for HF. MACE was defined as cardiovascular death, hospitalization for HF, and myocardial infarction. Both MACCE and MACE were used as composite endpoints.

### Cardiac magnetic resonance imaging

CMRI scans were performed with a Siemens MAGNETOM Avanto and Avanto fit 1.5 T scanner (Siemens Healthineers, Erlangen, Germany) using multi-channel receiver coil setup. All images were acquired using a standard cardiac imaging protocol during breath-hold and with electrocardiogram (ECG) gating. Cine-images were acquired using a balanced steady-state gradient echo (TrueFISP) sequence. All studies included standard 2-, 3-, and 4-chamber cine images. In addition, a stack of short axis images was obtained from the mitral valve plane to the apex. The imaging parameters were: echo time (TE) 1.07–1.23; flip angle 55–76°; image matrix 113–156 × 192; slice thickness 6–8 mm; and acquisition voxel size 1.4–1.8 × 1.4–1.8 mm^2^. The temporal resolution was 33.7–58.3 ms.

To detect myocardial scar, late gadolinium enhancement images were acquired with a 2D-segmented inversion recovery gradient echo sequence 12–20 min after gadolinium (Dotarem®, 279.3 mg/ml, dose 0.2 mmol/kg) injection. Inversion time was optimized for each measurement to null the signal intensity of normal myocardium (240–360 ms). Late gadolinium images were obtained at the same views and slice/gap thickness as cine imaging.

Images from all the previous data points (baseline, 1- and 5-years follow-ups) were retrieved from hospital radiology data archives. All images were analyzed using QMass and QStrain cardiac imaging analysis programs (Medis Medical Imaging Systems, Leiden, The Netherlands). Endocardial and epicardial contours of the left ventricle were semi-automatically annotated to all cine-series and short-axis contours were used to evaluate LV volumes, mass, and ejection fraction. Short-axis endocardial contours of the right ventricle were annotated to evaluate right ventricle (RV) volume and ejection fraction. Standard 2D IR sequence was used to evaluate late gadolinium enhancement. Signal threshold versus reference mean (STRM) threshold of 5 standard deviation (SD) above the mean signal intensity (SI) was used to assess myocardial and infarcted area mass to obtain the scar volume percentage from all the 16 LV segments as in our previous studies^2,3^.

The Δscar volume% was calculated from all the 16 LV segments^12^ by comparing scar volumes from each segment to different time points, segment—by—segment, to obtain the effect of injection in injected segments, and the changes in scar volume in noninjected segments of the cardiac tissue. Three independent researchers analyzed the CMRIs. First, two blinded researchers (SM, RK, both resident level) quantified the images. Data from their analyses showed interobserver agreement of 0.831 indicating good comparability. A CMRI specialist (MH) then checked each analysis for validity, and this data was then used for the final analysis of scar mass, scar volume, chamber volumes and LVEF. Even though MH was involved in the image analysis previously^2,3^, all attempts to ensure her blinding to the grouping were made.

### Statistical methods

All statistical analyses were done by using IBM SPSS Statistics (IBM’s SPSS Statistics for Windows, version 25.0, IBM Corp., Armonk, NY). Mann–Whitney U test was performed for continuous variables, with the results reported as medians with 95% confidence interval (CI). Categorical variables were analyzed with Fisher’s exact test. We estimated hazard ratios (HRs) with 95% CIs for all-cause-mortality, cardiovascular mortality, MACCE and MACE between groups. For this analysis, we used Cox proportional hazard model in IBM SPSS Statistics.

All *p*-value testing were two-sided, with statistical significance at *p* < 0.05. All the analyses were done blinded, and blinding was dismantled at the end of the study. Blinding was ensured so that the data analyzing researcher were kept blinded for the grouping. After all data analyses and statistics were completed, the group coding was released by the analyzer of the previous results (ML), who did not participate in the analysis of the current data.

## Results

**Figure 2:**
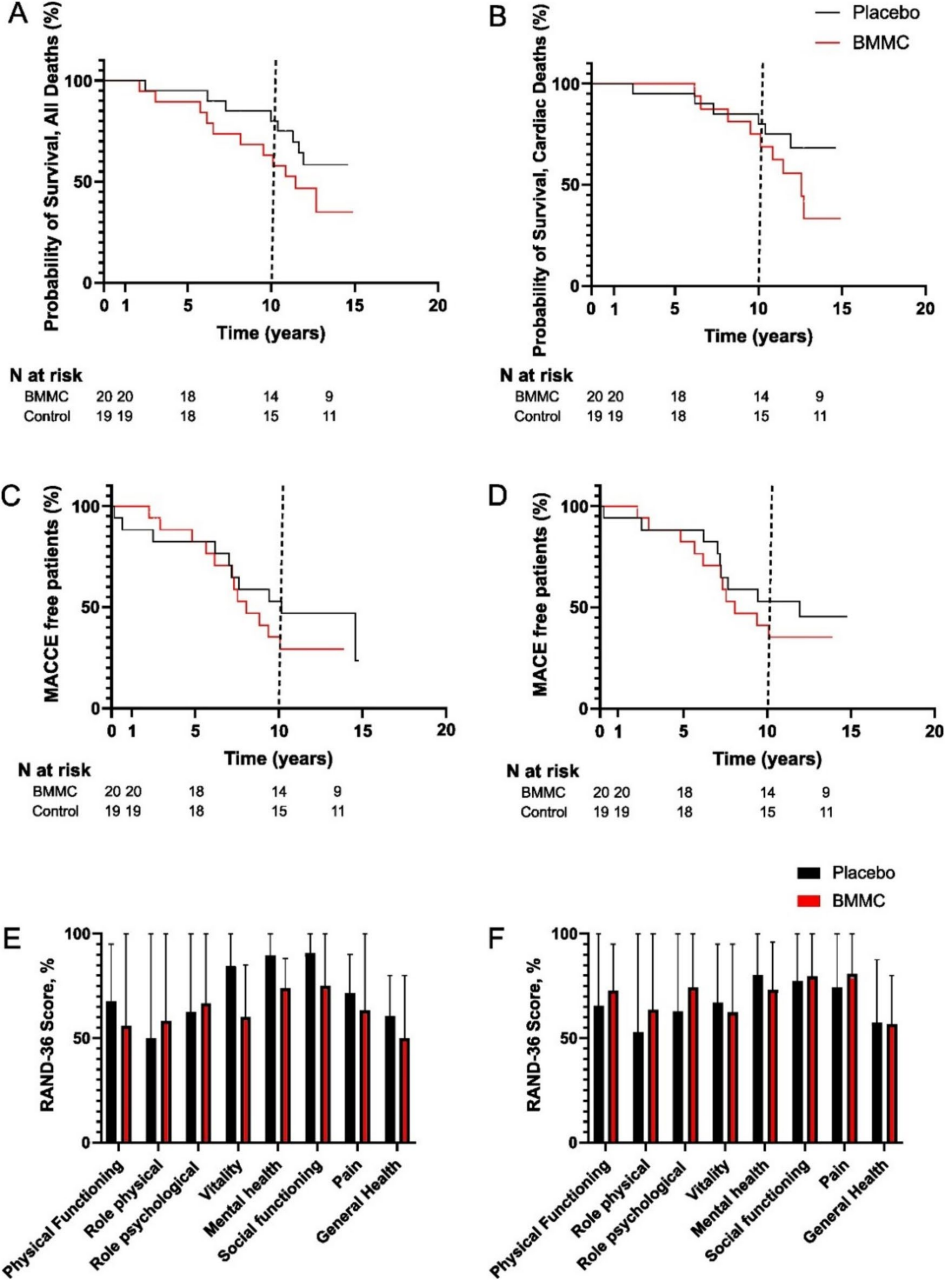
(**A**) Overall survival between groups during over the 10-year follow-up. (**B**) Survival from cardiac deaths, (**C**) MACCE-free patients during follow-up, and (**D**) MACE-free patients during follow-up. (**E**, **F**) Means and standard deviations (whiskers) of scores for different dimensions of the Medical Outcomes Study Short-Form 36 (SF-36) health related quality of life questionnaire for patients treated with BMMC or placebo therapy (**E**), 10-years time-point, (**F**), 5-years time-point). The vertical dashed line represent point after which the number of follow-up patients decreased due to variety of operation date.

Survival and mortality data were obtained from all the patients (N = 39). None of the patients died before 1-year follow-up. Between one-year follow-up and our previous follow-up in 2013, two patients from the BMMC-group and one patient from the control-group had died. During the current study’s evaluation period of more than 10-years follow-up, a total of 11 (55%) patients from the BMMC-group and seven (37%) patients from the control-group died. (Fig. 1). In the BMMC group, the primary reason for death was atherosclerosis for eight (40%) patients, renal carcinoma for one (5%) patient, pancreatitis for one (5%) and Alzheimer’s disease for one (5%) patient. In the control group, primary reason for death was atherosclerosis for six (32%) and prostate carcinoma for one (5%) patient. The all-cause mortality for the BMMC group did not differ from control group with a HR of 1.87 (95% CI 0.75–4.63, *P* = 0.170) nor did cardiovascular mortality with a HR of 2.03 (95% CI 0.73–5.66, *p* = 0.169) (Fig. 2A,B).

Of the original 39 patients participating in the study, 18 patients (46%) had died. Of the 21 patients alive, four (19%) declined participation in this follow-up (Fig. 1). Thus, the study groups in this follow-up consisted of eight patients in the BMMC groups and nine patients in the placebo group. Before closing this study for analyses but after all patients had undergone CMRIs, one more patient from the control-group died due to cardiovascular reason. The median follow-up time was 13 (range 10–15) years and the patients’ age in the BMMC group was 78 (range 69–87) and in the placebo group 75 (range 65–87) (Table 1).

**Table 1:** Patient characteristics, follow-up time and events during follow-up.

Variable	Placebo group	BMMC group	*P* value
Patients participating in study	9	8	–
Follow-up, median (years)	13 (11–15)	13 (11–15)	0.815
Age (years)	75 (65–87)	78 (69–87)	0.606
Male	8	7	1.000
Hospitalization due to HF	2	1	1.000
Myocardial Infarction	0	0	–
ICD or pacemaker	2	1	1.000
CABG or PCI	0	0	–
Stroke	1	2	0.576
Cancer	0	1	0.471
Six-minute walk test (m)	374 (275–480)	328 (100–505)	0.731
NT-ProBNP	1241 (307–3980)	3414 (378–9351)	0.200
GFRe (ml/min/1.73 m^2^)	67 (30–91)	75 (21–98)	0.423
fP-Cholesterol (mmol/l)	4 (2.5–5.1)	3.5 (2.6–4.3)	0.328
fP-LDL (mmol/l)	2.3 (1.0–4.5)	1.8 (1.2–3.0)	0.139
fP -HDL (mmol/l)	1.0 (0.7–1.7)	1.2 (0.9–1.9)	0.195
fP-Triglycerides (mmol/l)	1.7 (1.1–2.7)	1.4 (0.7–2.6)	0.279
B-HbA1c (mmol/mol)	43 (32–54)	53 (35–87)	0.328
Ejection fraction (%)	47 (35–62)	42 (20–58)	0.673
Medication			
ACE-inhibitor or AT1-blocker (%)	9 (100%)	8 (100%)	–
Diuretic (%)	3 (33%)	3 (38%)	1.000
Beta blocker (%)	8 (89%)	8 (100%)	1.000
Spironolactone (%)	1 (11%)	1 (13%)	1.000
Nitrate (%)	0 (0%)	1 (13%)	0.471
ASA (%)	6 (67%)	4 (50%)	0.637
Ca^2+^-channel blocker (%)	2 (22%)	2 (25%)	1.000
Statin (%)	7 (78%)	8 (100%)	0.471
Warfarin (%)	4 (44%)	0 (0%)	0.082
NOAC (%)	0 (0%)	4 (50%)	0.029*

Values in parentheses indicate range, min–max. * denotes statistical significance between groups at *P* < 0.05. Abbreviations: ACE, angiotensin-converting enzyme II; ASA, acetylsalicylic acid; AT1, angiotensin II receptor type 1; CABG, coronary artery bypass grafting; GFRe, glomerular filtration rate, estimated; HbA1c, glycated hemoglobin; HDL, high-density lipoprotein; ICD, implantable cardioverter defibrillator; LDL, low-density lipoprotein; NOAC, novel oral anticoagulant; fP, fasting plasma; NT-Pro-BNP, N-terminal fragment of pro-B-type natriuretic peptide; PCI, percutaneous coronary intervention.

MACCEs and MACEs were obtained from all the patients participating in the original study. There were no differences in MACCEs (HR = 1.447, 95% CI 0.625–3.349, *p* = 0.538) or in MACEs between groups (HR = 1.360, 95% CI 0.565 to 3.271, *p* = 0.434) (Fig. 2C,D). Moreover, no statistically significant differences were obtained in any dimension of HRQoL between groups at the 5-years follow-up or in the over-10-years follow-up (Fig. 2E,F).

Two patients in the control group had been hospitalized due to HF, and one patient in the BMMC group had a hospitalization period due to HF. Two patients from the control and one patient from the BMMC group had an implantable cardioverter defibrillator (ICD) or cardiac resynchronization therapy pacemaker implanted during follow-up. One patient in the BMMC group was diagnosed with cancer (sigmoid adenocarcinoma) during follow-up. Strokes occurred for two patients in the BMMC group and one from the control group. The patients’ serum cholesterol levels did not differ between groups (Table 1). No statistical significance between groups were observed in NT-ProBNP levels. At the timepoint of over 10 years the NT-ProBNP showed a non-significant trend of higher values in the BMMC group (3414; 95% CI 430–6400 vs. 1241; 95% CI 380–2100, *p* = 0.200) (Table 1).

There were no statistically significant differences in the 6MWT (control: 374 m; 95% CI 330–430 m vs. BMMC: 328 m; 95% CI 220–440 m, *p* = 0.731) or in the LVEF measured by echocardiography between groups (control: 47%; 95% CI 41–53% vs. BMMC: 42%; 95% CI 29–50%, *p* = 0.673). In both groups, the occurrence of atrial fibrillation increased similarly over the follow-up time. In all, there were 6 cases of atrial fibrillation in the control group (6/19, 31.6%) and 11 cases in the treatment group (11/20, 55%) (*P* = 0.647). Interestingly, over the long-term follow-up three patients in the control group developed a left ventricular thrombus or an aneurysm predisposing to it. One of the patients in the control group had very severe failure. In the treatment group, no ventricular aneurysms or thrombi were observed. Thus, 21% (4/19) vs 0% (0/20) of patients in the control and treatment groups, respectively, had severe disturbances in the function or structure of the left ventricle during long-term follow-up. For the patients participating to this long-term follow-up, there were 4 cases of atrial fibrillation in the control group (4/9, 44%) and 4 cases in the treatment group (4/8, 50%) (*p* = 0.888). This was also reflected in the frequent use of novel oral anticoagulant (NOAC)s as compared to warfarin in the BMMC group (Table 1).

**Figure 3:**
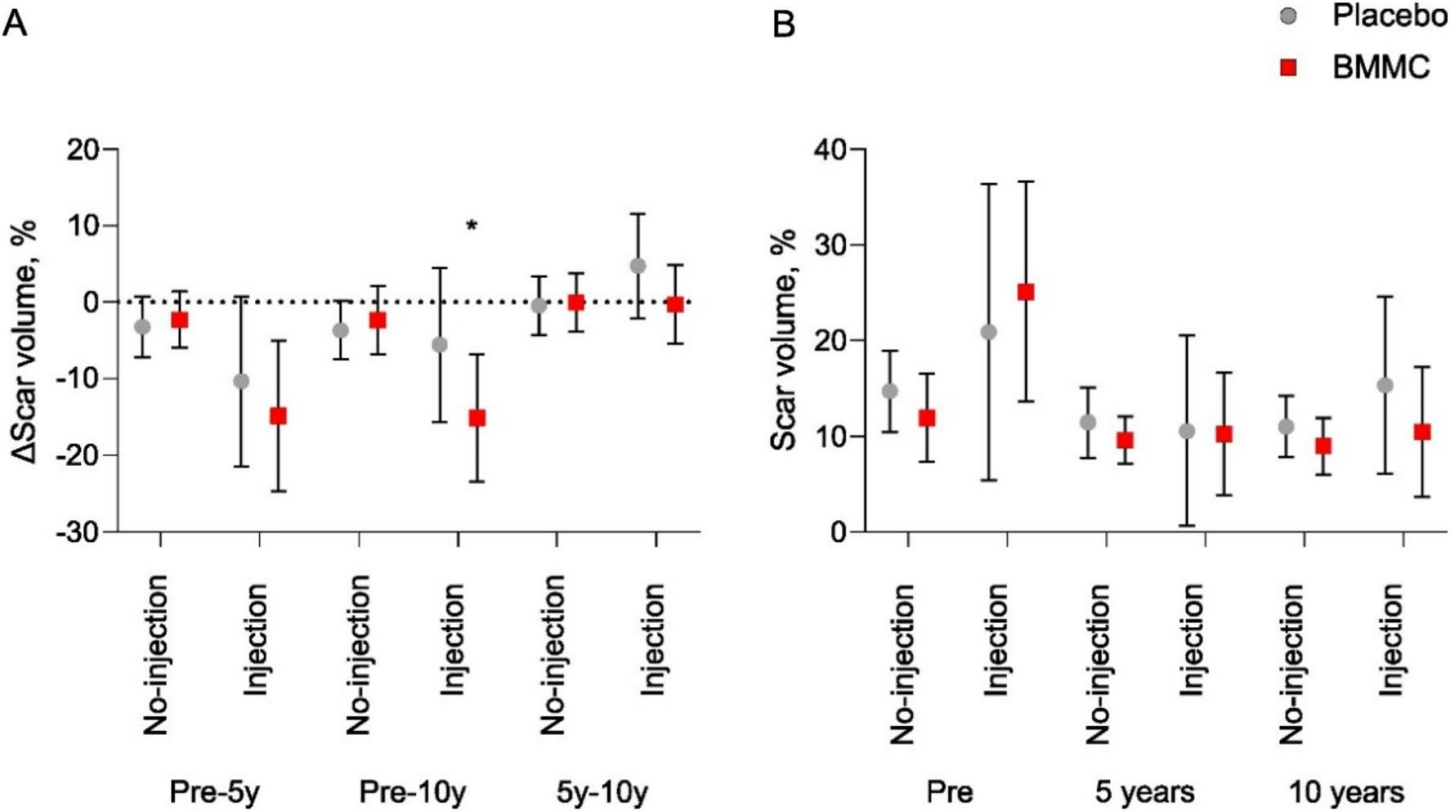
(**A**) Median scar volume changes with 95% CI (whiskers) during follow-up time between BMMC and placebo treated segments and (**B**) Median scar volumes. *Denotes statistical significance between Injection and No-injection segments (*P* < 0.05).

**Figure 4:**
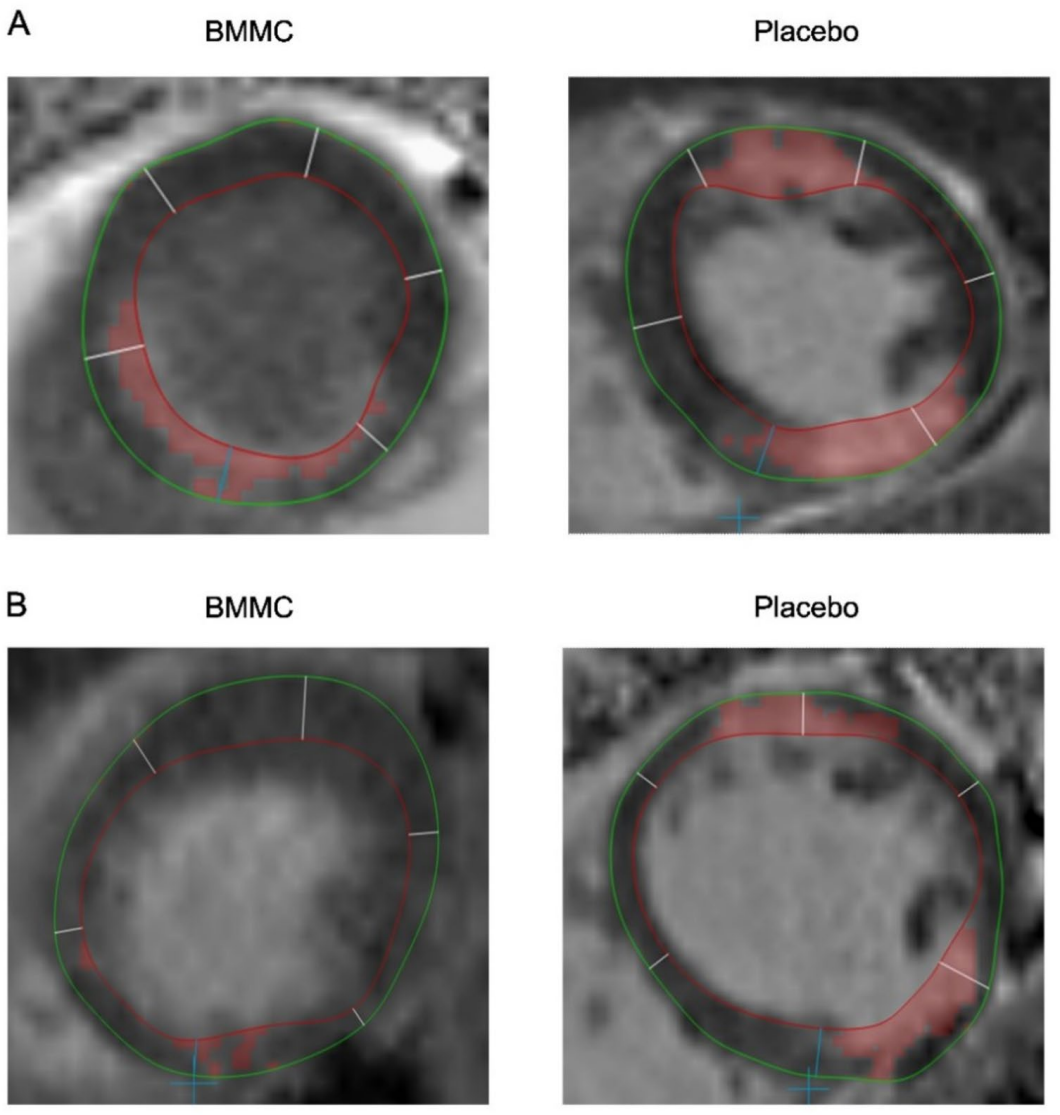
Demonstrating axial CMRI figures of pre- and postoperative scar volume percentages. (**A**) Preoperative scar volume percentages between groups, and (**B**) scar volume percentages after 10 years of follow-up. Green line represents epicardial contour, red line endocardial contour, and delicate red area represent scar tissue.

CMRIs were obtained for 14 patients (seven BMMC and seven control patients)—one BMMC patient and two control patients were withdrawn from CMRI due to condition (Fig. 1). Depending on the size and location of the myocardial scar, the infarction border area that received BMMC or placebo injections comprised 1–4 myocardial segments according to the topographic standardization and nomenclature by the American Heart Association^12^. The scar volume percentage and its changes in segments with and without injections for both BMMC and control groups are represented in Figs. 3 and 4 and in the Supplementary Table 2. In the segments receiving either BMMC or placebo injections, the changes in scar volumes demonstrated greater reduction in the BMMC group between the preoperative and the over 10-years follow-up values (BMMC: − 15.1%; 95% CI − 23 to − 6.7% vs. − 7.3%; 95% CI − 16 to 4.5%, *p* = 0.039) (Fig. 3A). In the myocardial segments receiving no injections, no statistically significant differences in scar volume changes were observed between groups (BMMC: − 2.3%; 95% CI − 6.8 to − 2.3% vs. control: − 3.7%; 95% CI − 7.5 to − 3.7%, *p* = 0.966). When analyzing the follow-up period between the 5-year time point and the over-10-years time point, we found no significant differences in scar reduction. Scar volume changes in the injected zones were 0.3% (95% CI − 5.4 to 4.9%) and 4.5 (95% CI − 2.0 to 12%) (*p* = 0.298) for the BMMC and control groups respectively. The result shows a non-significant, trend of increased scar formation in the control group. In the segments receiving no injections, the scar changes were similar (BMMC: − 0.1%; 95% CI − 3.9 to 3.8% vs. control: − 0.5%; 95% CI − 4.3 to 3.4%, *p* = 0.687). Overall, at the over-10-years time point, the scar volumes were not significantly different in the injected segments (BMMC: 10.3%; 95% CI 3.7–17% vs. control: 16.5%; 95% CI 6.1–25%, *p* = 0.239) or in the segments receiving no injections (BMMC: 8.9%; 95% CI 6.0% to 12% vs. control: 11%; 95% CI 7.8–14%, *p* = 0.312) (Fig. 3B). The overall scar mass (g) did not differ between groups preoperatively (BMMC: 7.9; 95% CI 4.8–11.3 vs. control: 10.0; 95% CI 6.4–14.9, *p* = 0.699), or after 10-years of follow-up (BMMC: 3.9; 95% CI 0.5–7.4 vs. control: 4.0; 95% CI 0.6–11.3, *p* = 0.589).

**Figure 5:**
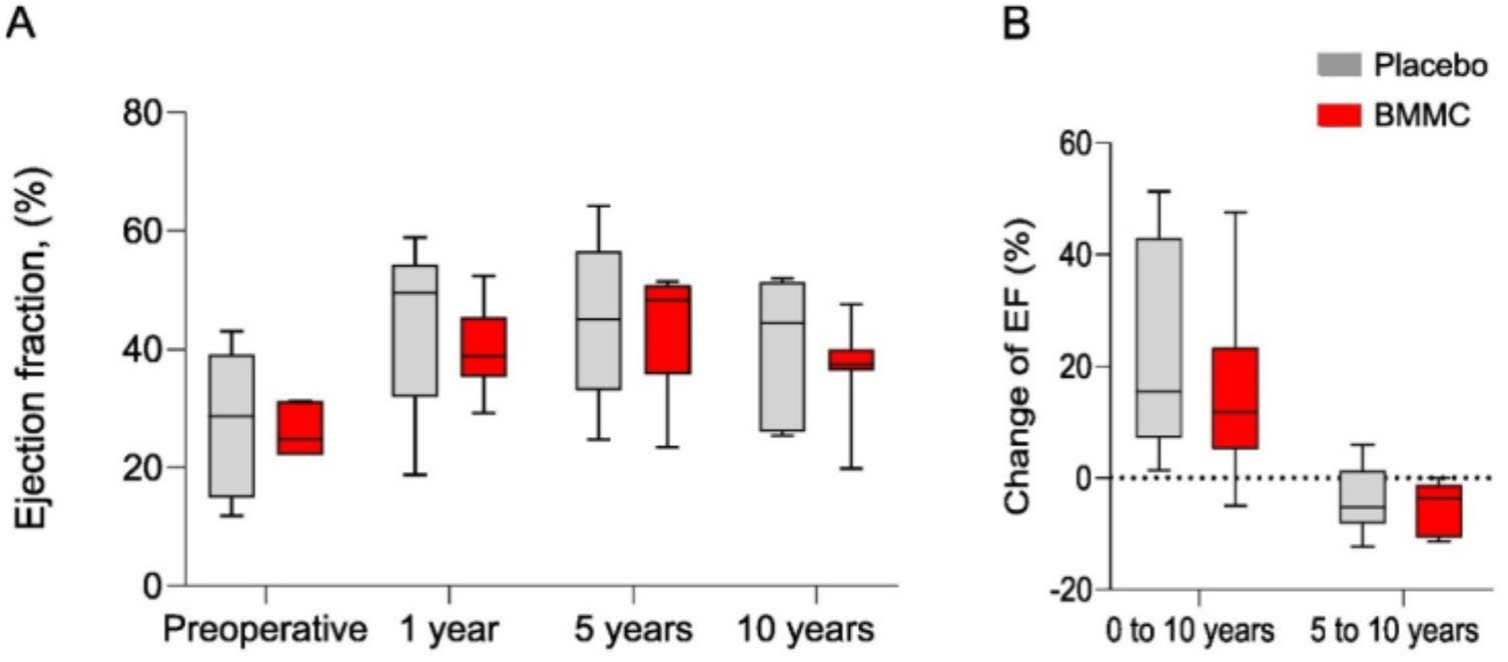
Ejection fractions (EF) (**A**), EF changes (**B**) during follow-up time between BMMC treated and control patients. The horizontal line in the middle of each box indicates the median, the box borders indicate the 25th and 75th quartiles, and the whiskers indicate the range. Abbreviations: EDV, end-diastolic volume; EF, ejection fraction; ESV, end-systolic volume.

**Figure 6:**
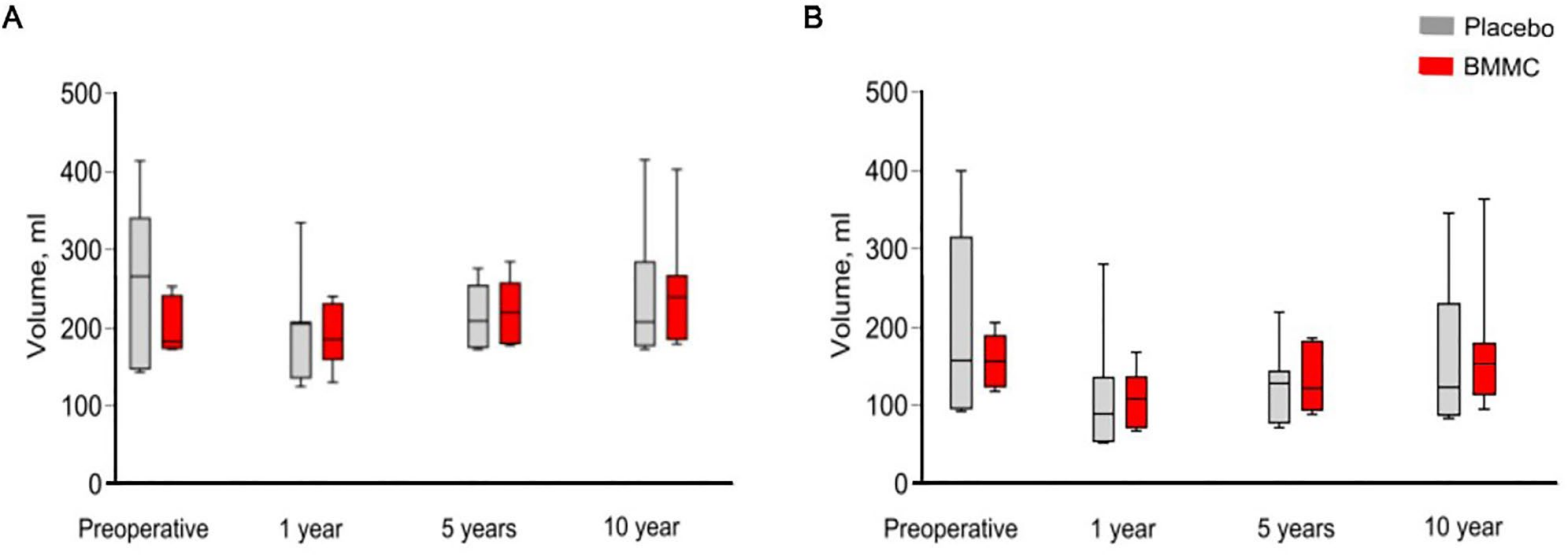
Left ventricle end diastolic (LVEDV) (**A**) and end systolic volumes (LVESV) (**B**) during follow-up time between BMMC treated and control patients. The horizontal line in the middle of each box indicates the median, the box borders indicate the 25th and 75th quartiles, and the whiskers indicate the range.

LVEF assessed from CMRI data, and its change are presented in Fig. 4A,B. There were no statistically significant differences in LVEF between groups during follow-up time (Fig. 5A). Change of LVEF (Fig. 5B) between preoperative and over 10-year follow-up did not reach statistical significance between groups: control patients (22.3%; 95% CI 5.2%–39%) vs. BMMC (15%; 95% CI − 3.4 to 34%), *p* = 0.481. Reduction of LVEF between five and over 10-year time points remained not statistically significant as well. Left ventricle end diastolic volumes (LVEDV) and LVESV during follow-up are presented in Fig. 6. No statistically significant differences between groups were observed. The total LV mass during follow-up is presented in Supplementary Table 3. No statistically significant differences were observed, in any time-point, between groups.

## Discussion

We present here the results of our long-term, spanning a period of over 10 years, follow-up study of patients with ischemic heart failure enrolled during 2006–2011 in the Helsinki BMMC Collaboration trial. The trial encompassed 20 patients receiving BMMC injections and 19 patients receiving placebo injections at CABG surgery. The study was specifically designed to address the shortcomings of other BMMC studies, and was carried out in a randomized, placebo-controlled manner on patients remaining refractory to drug treatment over a preoperative period of heart failure medical therapy optimization. These patients have been earlier evaluated both clinically and using CMRI at one-year and five-year follow-ups^2,3^. Comparing the cardiac segments receiving either BMMC or placebo injections, we have previously shown that the early improvement of myocardial scar by BMMC therapy persists at the five-year follow up. In the current study, we utilized state-of-the-art CMRI analysis techniques, and both re-evaluated the previous results and newly imaged the hearts of patients alive.

Our results, at this over-10-years follow-up, demonstrated greater reductions in scar size specifically in those patients and cardiac segments having received BMMC injections as compared to patients and cardiac segments receiving placebo injections. These results suggest that BMMC treatment of ischemic heart failure administered intramyocardially in conjunction with CABG surgery may exert unprecedented long-term scar-reducing effects. However, we observed no difference between groups in mortality or LVEF in this limited size study.

The results are in line with our previous findings; in one-year follow-up, we noticed a 13.1% reduction in scar size of BMMC-injected segments. In comparison, scar size increased by 5.1% in placebo-treated segments^2^. In the five-year year follow-up, the scar volumes of BMMC-treated segments reduced by 17% while in placebo-treated segments the scar volumes increased by 2%^3^. After current follow-up, the scar size of injected segments remains same in BMMC treated segments—unlike previously, now we obtained small scar size reduction in placebo treated segments also. Apparently, the therapy fails to affect volumes and systolic function assessed by CMRI, because there were no differences in any of these parameters during our 1-, 5- or the current over 10-years follow-up. No differences were obtained in mortality, MACCEs, HRQoL or 6MWT between groups after 10 years of follow-up.

Some recent meta-analyses have summarized the effect of BMMC therapy in long-term follow-up; in 2016, Lee et al. concluded that cell injection decreased all-cause mortality, but overall scar size did not differ in three- or five-year time points measured by CMRI^10^. This meta-analysis included 43 RCTs, where most cell injections were performed through intracoronary infusion and 2–7 days after percutaneous coronary intervention and thus doesn’t fit properly to make conclusions about intramyocardial BMMC delivery during CABG. Another meta-analysis, conducted by Wang et al. summarized results of 14 RCTs studying effects of intramyocardial BMMC treatment^9^. They concluded that treatment did not affect mortality, but the treatment group had improvement of LVEF, LVESV, and NYHA and CCS classes. The follow-up period of these studies varied between 2 and 60 months, and thus doesn’t provide information about effects of treatment over a 10-year period.

Safety of BMMC treatment has been previously shown by us^11^ and other studies^13^, and some studies have even shown reduced mortality in BMMC treated patients^8^. Yet, the trend of mortality, MACCEs or MACEs are not favorable for the treatment group—although none of these parameters resulted statistically significant. The study population participating to the original study was relatively low, and further follow-up studies are needed. Moreover, recent Swedish study studied mortality after CABG or PCI between 1 day to 10 years postoperatively in patients with ischemic heart disease and concluded that the probability of death at the 10-year time point was slightly over 50%^14^, matching our death rate of 55% in the BMMC group.

Myocardial scar is a known substrate for ventricular arrhythmias and myocardial fibrosis is associated with elevated mortality^15–17^. We showed reduction of scar volume in injected segments: however, in our study there were no differences between groups in prevalence of arrhythmias or mortality during follow-up time. Our study has limitations. The total number of patients participating to the original study was relatively low and decrease of patients during follow up time reduces analytical power of the study. Thus, for controlling any survivorship bias, opening the extended, long-term evaluation of the effects of BMMC transplantation are warranted from the many similar studies carried out worldwide. Moreover, our results further underscore the need to include such long-term evaluations in the design of cardiac stem cell trials^5,18^. The mortality data was retrieved from the national population archives and as such did not provide detailed information about the cardiovascular disease severity or comprehensive details regarding cardiac mortality, and the status of myocardial scar fibrosis in death patients remains unknown.

More than 1,000 patients have received BMMC therapy for heart failure in a number of different clinical studies^19,20^ but still no consensus exists on their efficacy and as such they have not been adopted as true clinical treatment options—even for a select group of patients. In fact, current studies are now designed to improve their effects, for example by intensifying drug treatments^21^. Nevertheless, the large number of patients having received BMMCs during two decades of research efforts should not be taken lightly. In contrast, the studies so far conducted are to be considered a true treasure chest and studies should now be conducted to evaluate the long-term effects of these treatments. Here, we addressed the results of the Helsinki BMMC Collaboration trial on patients receiving either placebo or BMMC intramyocardial injections. We demonstrated that intramyocardially injected BMMCs may exert long-term benefits or cardiac protection in patients suffering from ischemic heart failure. This long-term protective efficacy deserves further evaluation in patients having received BMMC therapy as parts of numerous international clinical studies conducted already over two decades.

### Supplementary Information

Supplementary Tables.

## Data Availability

The datasets used and analyzed during the study are available from the corresponding author on reasonable request.
