# Supplementary material for: Follow-up of intramyocardial bone marrow mononuclear cell transplantation beyond 10 years
Source: Sci Rep. 2024 Feb 14;14:3747. doi: 10.1038/s41598-024-53776-9 (PMC10866866; doi:10.1038/s41598-024-53776-9)
Supplement: Supplementary file 1 — Supplementary Tables. [file 41598_2024_53776_MOESM1_ESM.pdf]

Follow-up of intramyocardial bone marrow mononuclear cell transplantation beyond 10 years

Severi Mulari[1,2], Risto Kesävuori[3], Juhani A. Stewart[1], Pasi Karjalainen[1], Miia Holmström[3], Miia Lehtinen[1], Juha Peltonen[3], Mika Laine[1], Juha Sinisalo[1], Tatu Juvonen[1], Markku Kupari[1], Ari Harjula[1], Tommi Pätilä[4], Sari Kivistö[3], Esko Kankuri[2,*], Antti Vento[1] on behalf of the Helsinki BMMC Collaboration

Supplementary Table 1. Exclusion criteria for patients participating in the original study.

**Exclusion criteria**
1. Heart failure due to left ventricular outflow tract obstruction
2. History of life-threatening ventricular arrhythmias or resuscitation, or insertion of implantable cardioverter-defibrillator
3. Stroke or other disabling condition within 3 months before screening
4. Severe valvular disease
5. Other severe disease limiting life expectancy
6. Contraindications for coronary angiogram or late gadolinium enhancement cardiac magnetic resonance imaging
7. Participation in other clinical trial.

Supplementary Table 2. Scar volumes and scar volume changes during follow-up time between groups. Reported as mean and range (min–max). Statistical differences were calculated using Mann-Whitney U test. * denotes statistical difference at *P*<0.05 between groups.

**Variable**	**Placebo group**	**BMMC group**	***P* value**	
Scar volume preoperative, %				
No injection	14.7 (1.1–85.7)	11.9 (0.9–79.1)	0.150	
Injection	23.9 (1.6–91.7)	25.1 (2.2–90.2)	0.284	
Scar volume 5 years, %				
No injection	11.5 (1.9–83.5)	9.6 (1.3–62.9)	0.301	
Injection	12.0 (2.6–73.5)	10.0 (1.8–66.1)	0.759	
Scar volume 10+ years, %				
No injection	11.0 (1.4–72.8)	8.9 (1.7–79.1)	0.312	
Injection	16.5 (2.8–61.5)	10.3 (2.5–70.3)	0.239	
ΔScar volume, %				
0 to 5 years				
No injection	−3.2 (−63.2–57.2)	−2.3 (−54.9–34.1)	0.477	
Injection	−11.9 (−76.0–3.6)	−14.8 (−75.1–6.6)	0.510	
0 to 10+ years				
No injection	−3.7 (−65.9–52.2)	−2.3 (−59.3–57.7)	0.966	
Injection	−7.3 (−52.5–17.6)	−15.1 (−71.6–11.4)	0.039	*
5 to 10+ years				
No injection	−0.5 (−59–70)	−0.1 (−37.2–56.3)	0.687	
Injection	4.5 (−15.6–36.4)	0.26 (−24.5–25.5)	0.298	

Supplementary Table 3. Total left ventricle (LV) mass (g) during follow-up time between groups. Reported as mean and range (min–max).

Total LV Mass (g)	Placebo group	BMMC group	*P* value
Preoperative	120.0 (86.7 – 162.3)	102.5 (67.0 – 131.4)	0.429
1 year	122.6 (82.2 – 147.7)	111.1 (84.9 – 135.2)	0.394
5 years	108.6 (88.0 – 133.6)	120.2 (85-4 – 149.1)	0.589
10 years	105.2 (86.4 – 115.8)	122.5 (89.8 – 143.5)	0.240

Acknowledgements

Collaborators in the Helsinki BMMC Collaboration group: Pekka Hämmäinen, Department of Cardiothoracic Surgery, Heart and Lung Center, Helsinki University Central Hospital; Jukka Schildt, Aapo Ahonen, and Päivi Nikkinen, Division of Nuclear Medicine, HUS Medical Imaging Center, Helsinki University Central Hospital; Anne Nihtinen, Department of Hematology, Helsinki University Central Hospital; Riitta Alitalo, Stem Cell Laboratory, Department of Clinical Chemistry and Hematology, HUSLAB, Helsinki University Central Hospital; and Reino Pöyhiä, Department of Anesthesiology and Intensive Care, Helsinki University Central Hospital, Helsinki, Finland.
